# Supplementary material for: Novel Metallo-Supramolecular Polymers with 1-Thioxophosphole Main-Chain Units and Remarkable Photoinduced Changes in Their Resonance Raman Spectra
Source: Polymers (Basel). 2022 Nov 30;14(23):5207. doi: 10.3390/polym14235207 (PMC9739564; doi:10.3390/polym14235207)
Supplement: Supplementary file 1 [file polymers-14-05207-s001.zip › polymers-1979571-supplementary.pdf]

Novel Metallo-Supramolecular Polymers with 1-Thioxophosphole Main-Chain Units and Remarkable Photoinduced Changes in Their Resonance Raman Spectra

Ivana Sloufova [1], Tereza Urvalkova [1,2], Muriel Hissler [2] and Jiri Vohlidal [1,]*

[1] Charles University, Faculty of Science, Department of Physical and Macromolecular Chemistry, Hlavova 2030, 128 40 Prague 2, Czech Republic

[2] University Rennes, CNRS, ISCR - UMR 6226, 35000 Rennes, France

Correspondence: vohlidal@natur.cuni.cz; sloufovi@natur.cuni.cz; muriel.hissler@univ-rennes1.fr

In the Raman spectra excited into MLCT band, the bands of phosphole units reversibly decay.

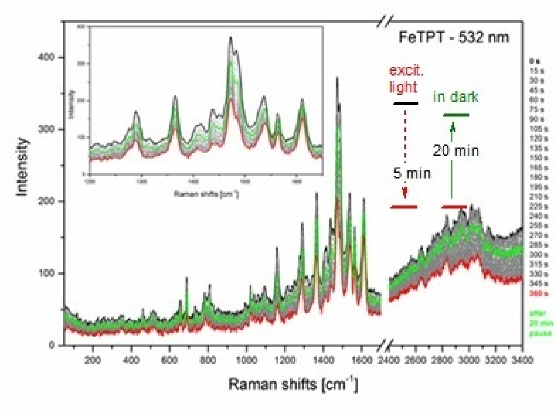

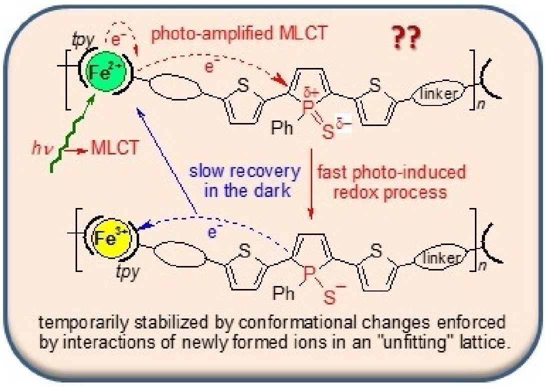

Supplementary Information

Table of contents

page

Figure S1. ^{1}H NMR spectra of prepared unimers (measured in CD_2Cl_2). 2
Figure S2. ^{13}C NMR spectra of prepared unimers (measured in CD_2Cl_2. 3
Monitoring of Zn-MSP and Fe-MSP assembly in solution 3
Figure S3. Changes in absorption spectra accompanying a stepwise assembly of MSPs 4-5
Figure S4. The UV/vis and photoluminescence solution spectra of unimers. 6
Figure S5. The UV/vis and photoluminescence solution spectra of MSPs. 6
Figure S6. The unimer luminescence quenching in response to the unimer binding to Fe^{2+} ions. 7
Figure S7. Time evolution spectral sets of the resonance (532 nm, 0.1 mW) and off-resonance (780 nm, 5 mW) Raman spectra; accumulation time 15 s; total time 6 - 9 min. 8
Processing of Raman spectral sets by Factor analysis (FA) 9
Figure S8. Results of factor analysis of time evolution spectral set of FeET, excitation 532 nm. 9
Figure S9. Raman spectral set for **FeEPh** (532 nm, **0.1 mW**) and the set after subtraction of fluorescence background to better see changes in spectral pattern. 10
Figure S10. Deconvolution of the second and next-to-the-last Raman spectrum of **FeEPh** and kinetics of obtained bands. 10
Figure S11. Overlaid baseline corrected spectra of the spectral sets of indicated compounds, normalized to the most intense spectral band 11

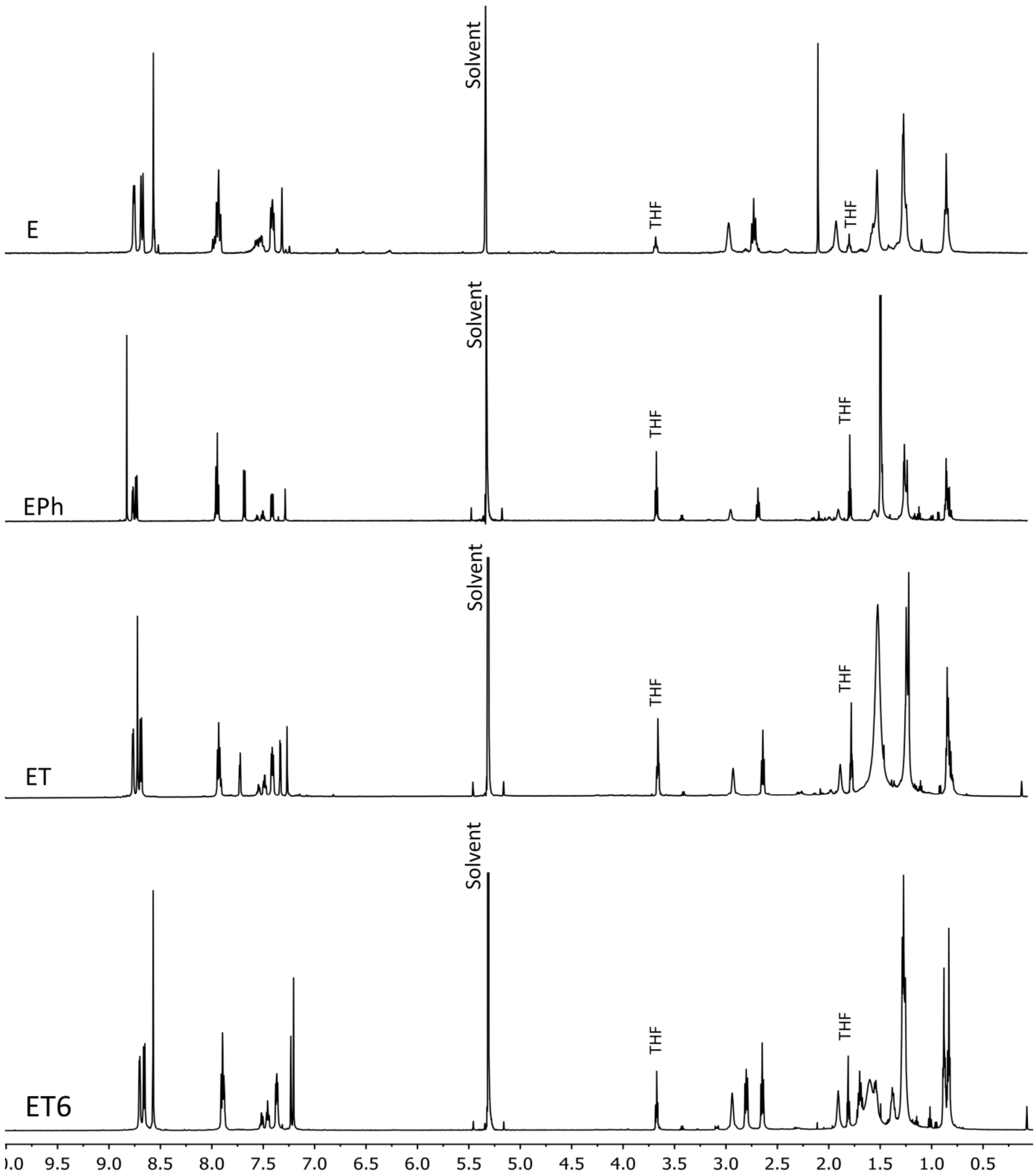

Figure S1. ^{1}H NMR spectra of prepared unimers (measured in CD_2Cl_2.

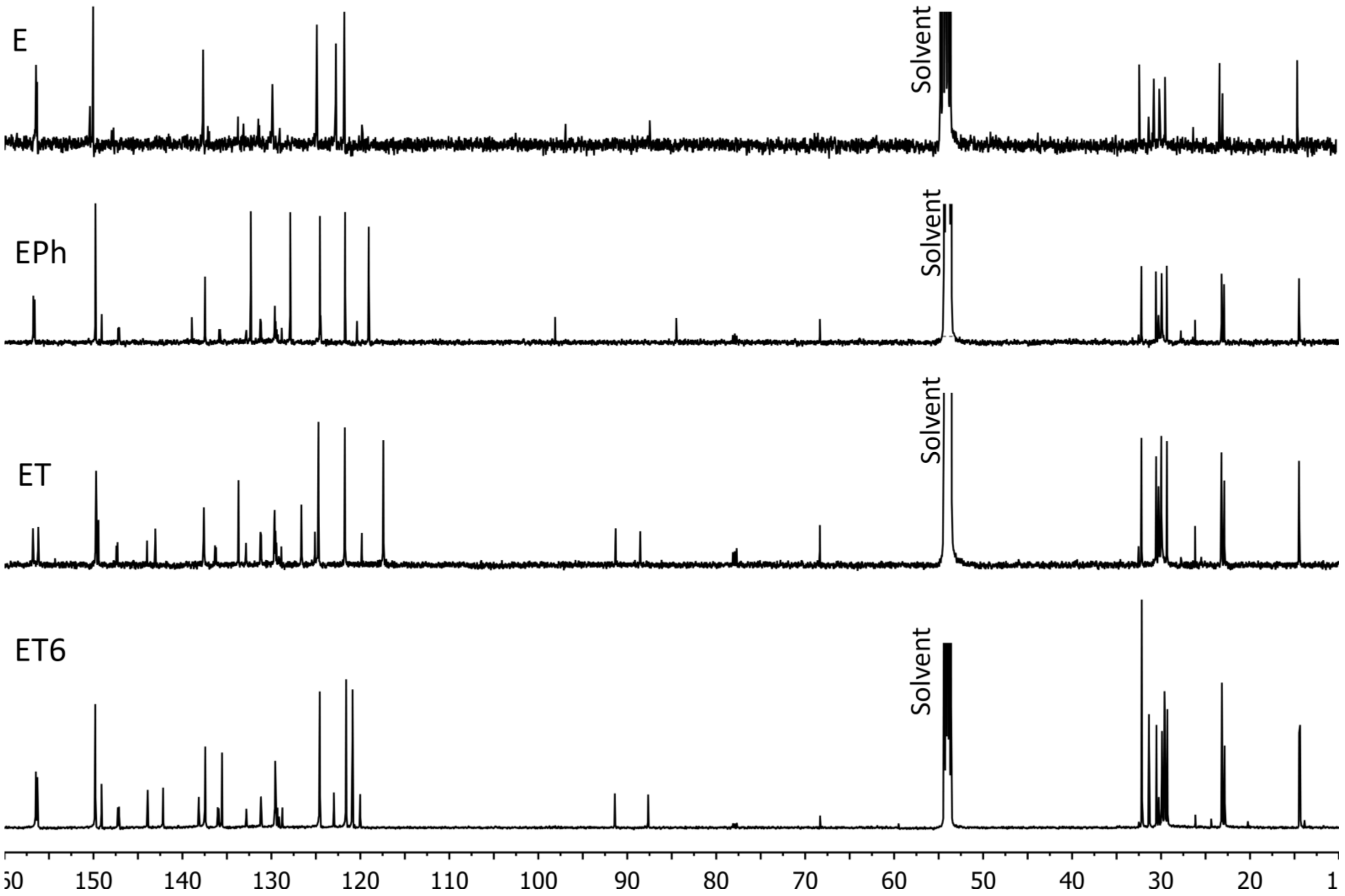

Figure S2. ^{13}C NMR spectra of prepared unimers (measured in CD_2Cl_2.

Monitoring of Zn-MSP and Fe-MSP assembly in solution

A measured volume of a solution (2×10^{-3} M) of Zn^{2+} or Fe^{2+} perchlorate in chloroform/acetonitrile mixed solvent was added into a unimer (**U**) solution (2×10^{-5}M) in the same solvent. The metal to unimer (M^{2+}/**U**) mole ratio: $r = M^{2+}$/**U** was varied from 0 to 3. The UV/vis absorption and the photoluminescence emission spectra of solutions were measured at room temperature one day after preparation.

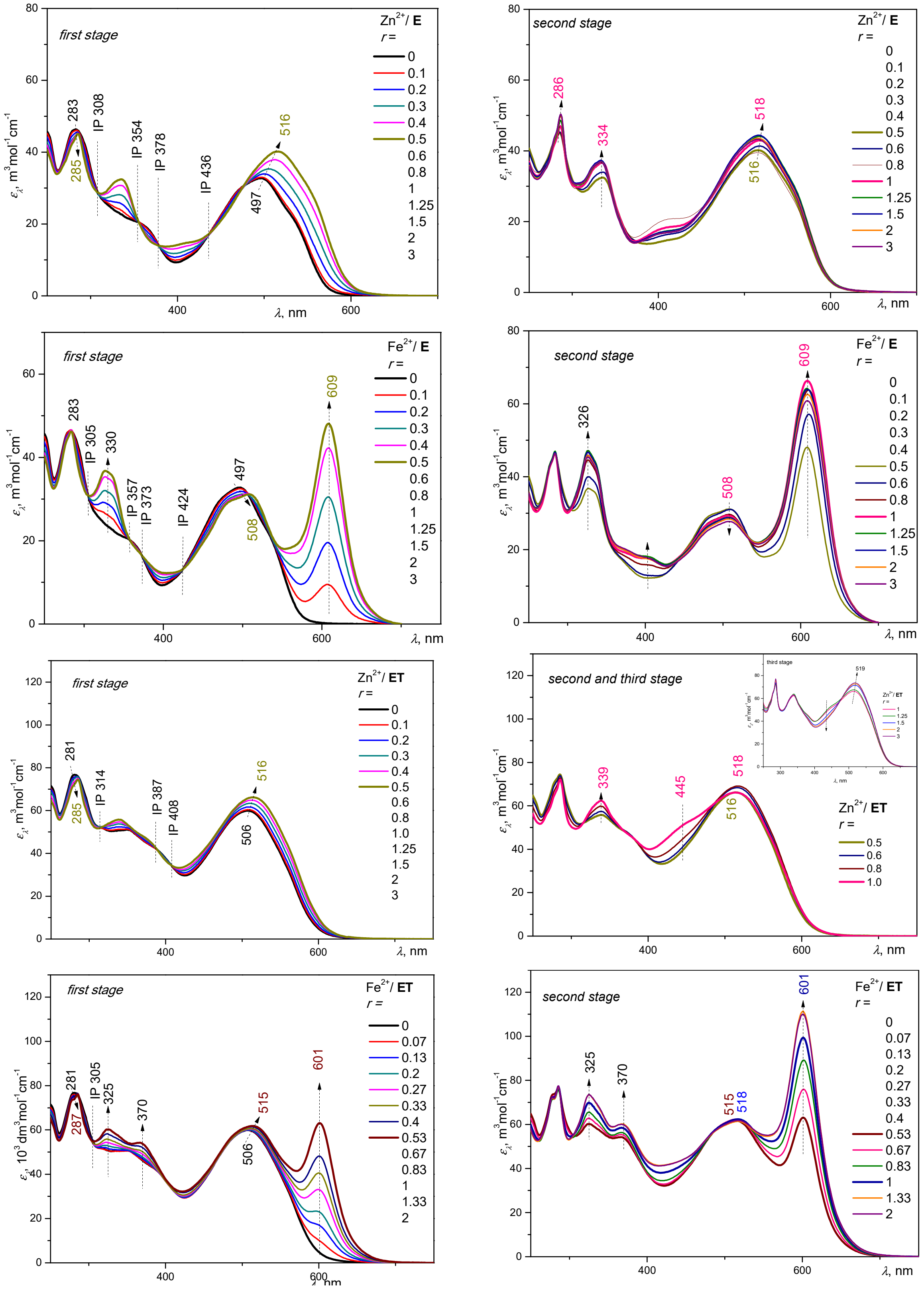

Figure S3-a. Changes in absorption spectra accompanying a stepwise assembly of MSPs from unimers **E** and **ET**.

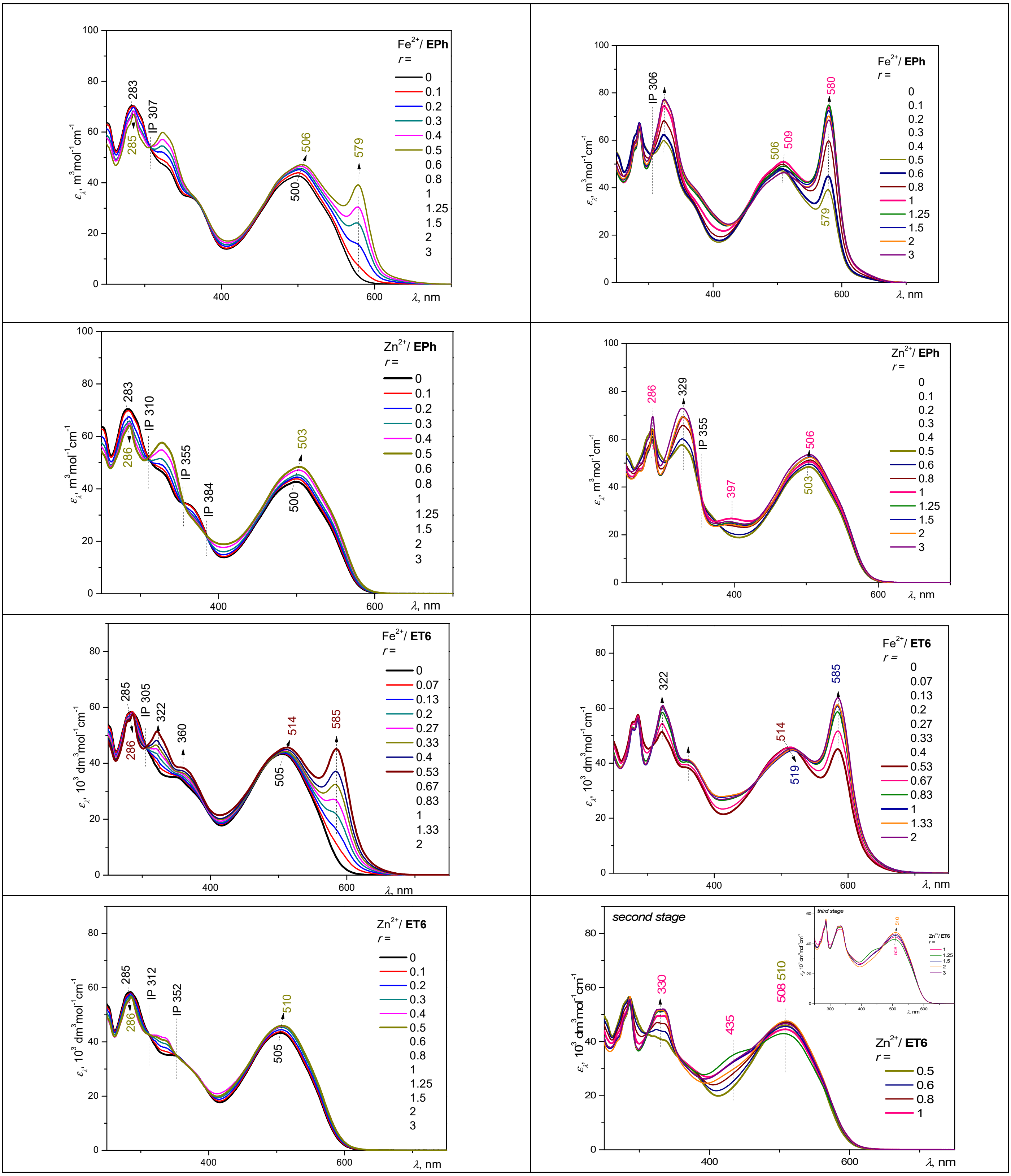

Figure S3-b. Changes in absorption spectra accompanying a stepwise assembly of MSPs from unimers **EPh** and **ET6**.

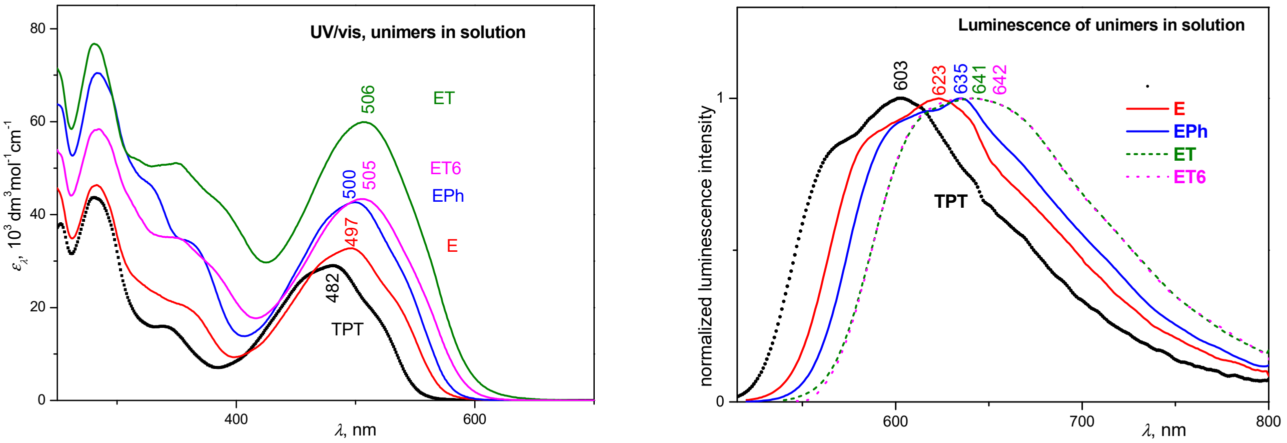

Figure S4. The UV/vis and photoluminescence solution spectra of unimers

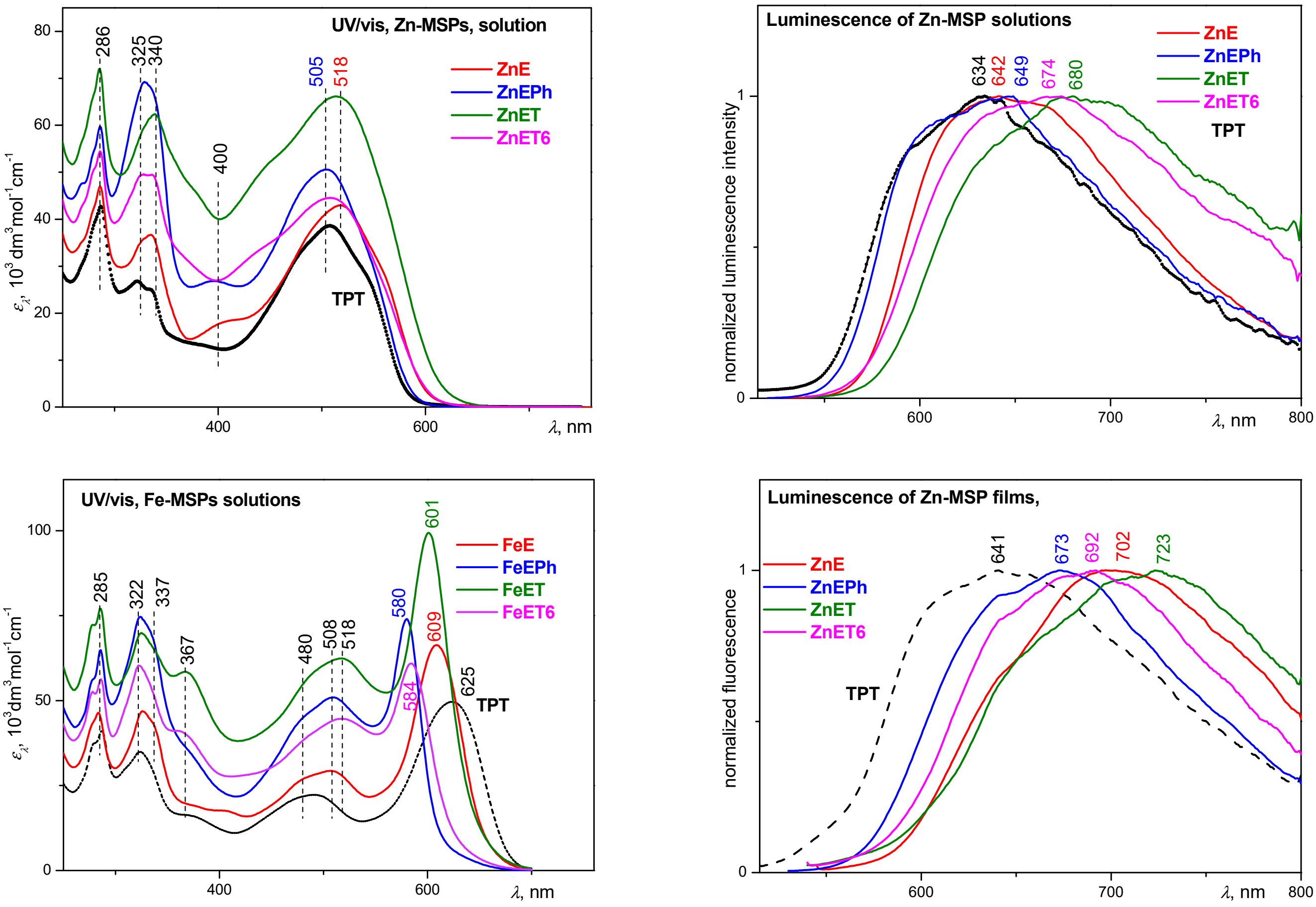

Figure S5. The UV/vis and photoluminescence spectra of MSPs; (Fe-MSPs are not luminescent).

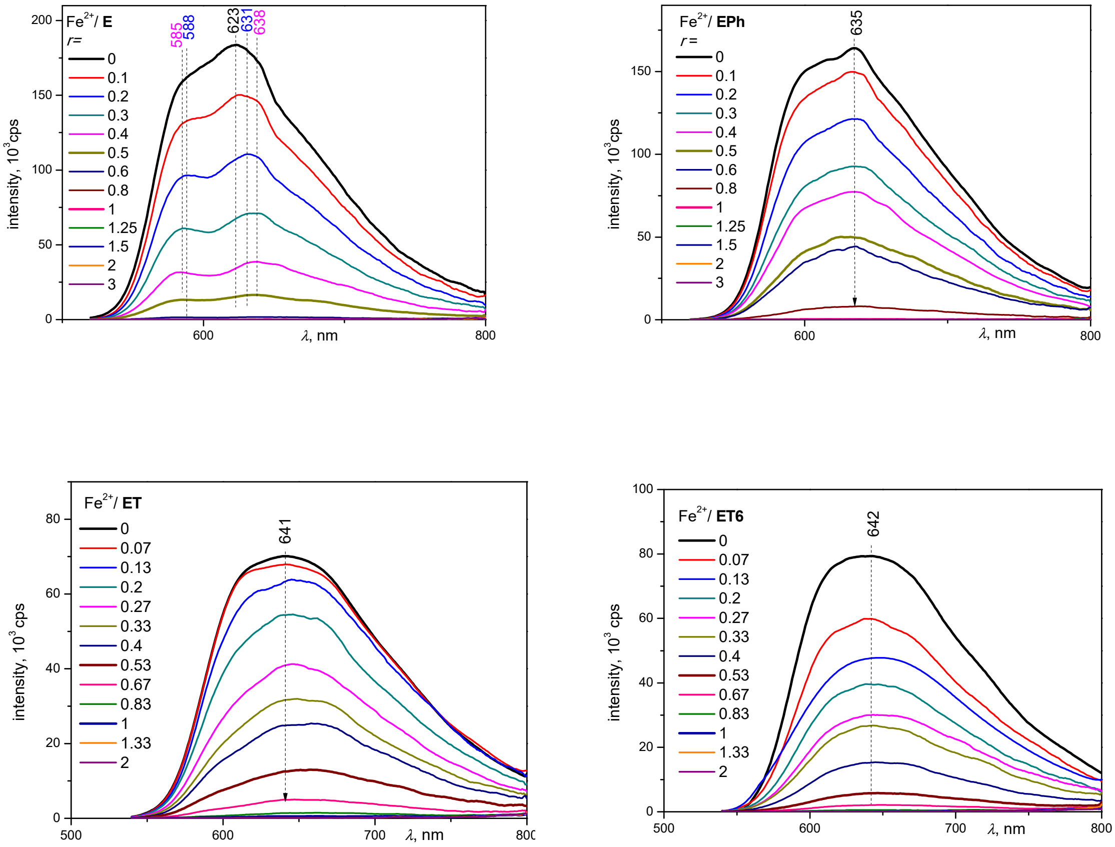

Figure S6. The unimer luminescence quenching in response to the unimer binding to Fe^{2+} ions. The luminescence totally disappears when the ratio *r* approaches the value of 1.

Thanks to the quenched luminescence, the resonance Raman spectra of Fe-MSPs can be easily measured, which is impossible in the case of ZnMSPs.

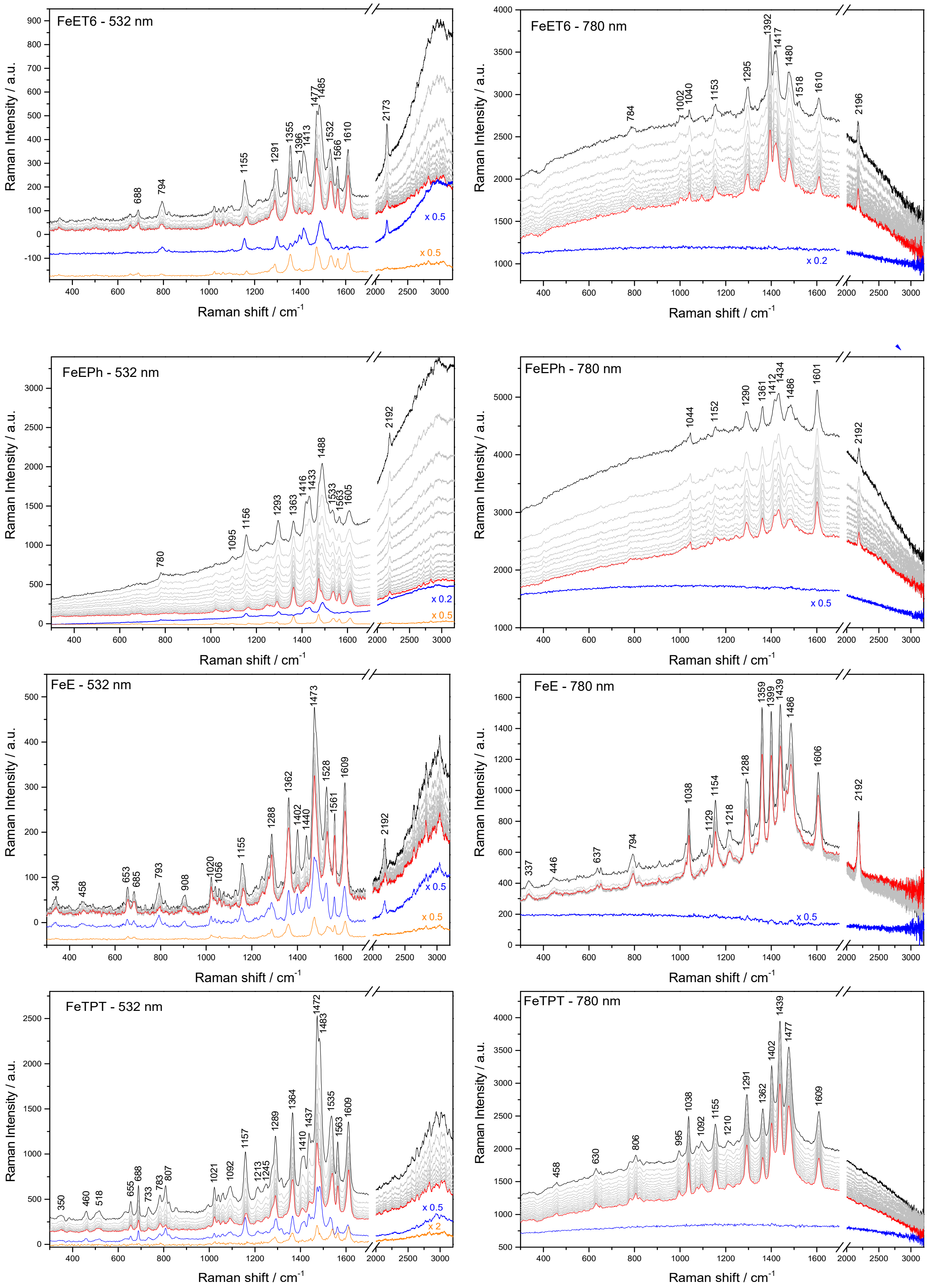

Figure S7. Time evolution spectral sets of the resonance (532 nm, 0.1 mW) and off-resonance (780 nm, 5 mW) Raman spectra; accumulation time 15 s; total time 6 - 9 min. (For **FeET** see the main part.

Processing of Raman spectral sets by Factor analysis (FA)

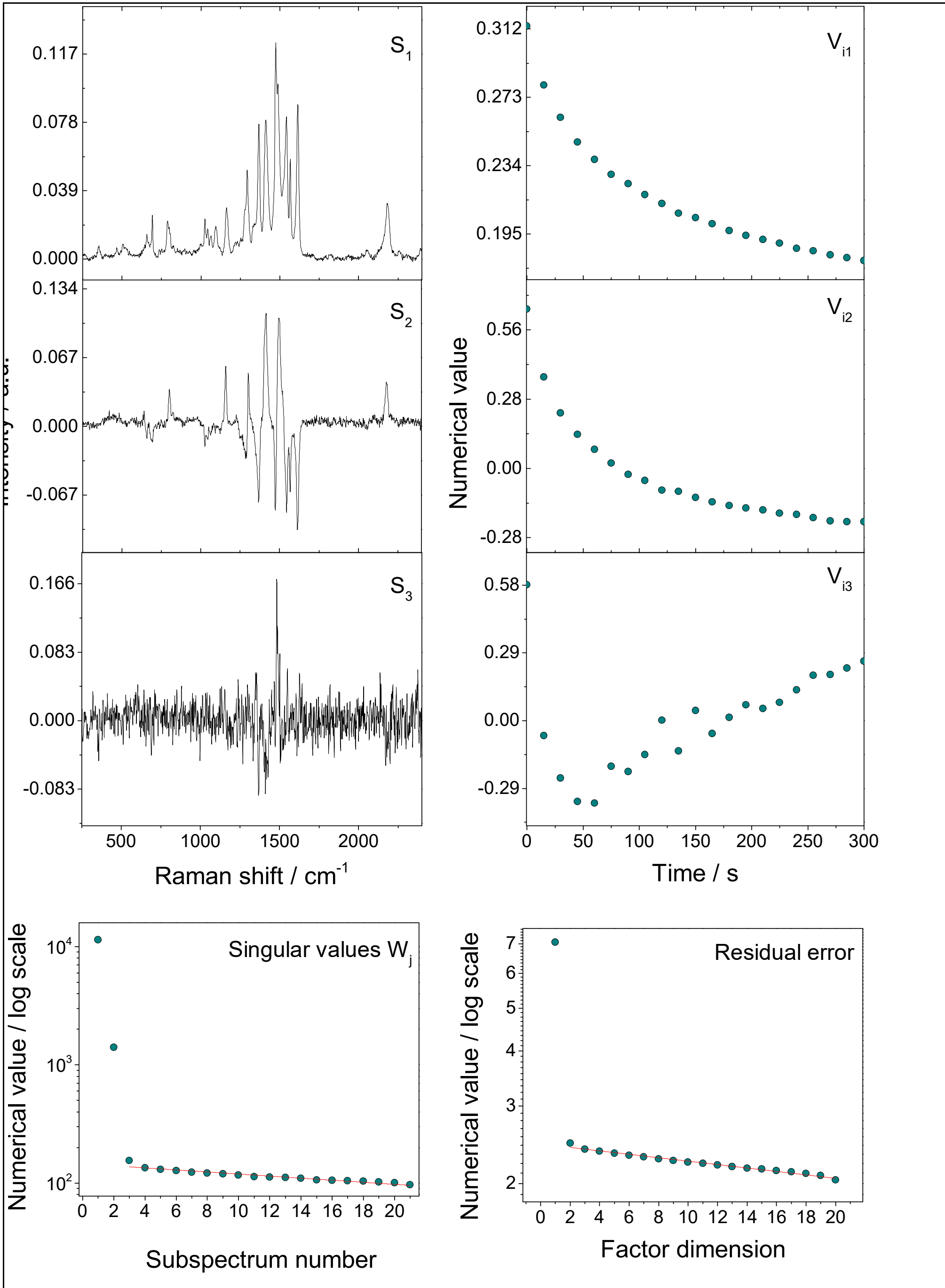

Spectral background of all spectra were corrected by the orthogonal differences method and then the whole spectral set was treated by factor analysis using the algorithm of singular value decomposition.

The FA procedure provides matrices of orthonormal subspectra $\boldsymbol{S_j}$ and coefficients $\boldsymbol{V_{ij}}$, and singular numbers $\boldsymbol{W_j}$. Each experimental spectrum $Y_i(\nu)$ of the set can be then approximated as:

$$Y_i(\nu) = \sum_{j=1}^{M} V_{ij} W_j S_j(\nu)$$

The factor dimension M (the number of subspectra needed for this approximation), is obtained from the break of the plot of singular numbers $\boldsymbol{W_j}$ vs the subspectrum number. Only the first two subspectra were found significant, as can be seen (bottom, left). The W_j values of higher subspectra were about order of magnitude lower and correspond to a noise (see subspectrum S_3). A sharp break at M = 2 on the dependence of the residual error on M (bottom, right) confirms correctness of this estimate.

A proper linear combination of the 1st and 2nd subspectrum so that no negative peaks were observed gives spectra of pure components involved in the monitored changes that are presented in the main text.

Figure S8. Results of factor analysis of time evolution spectral set of FeET, excitation 532 nm.

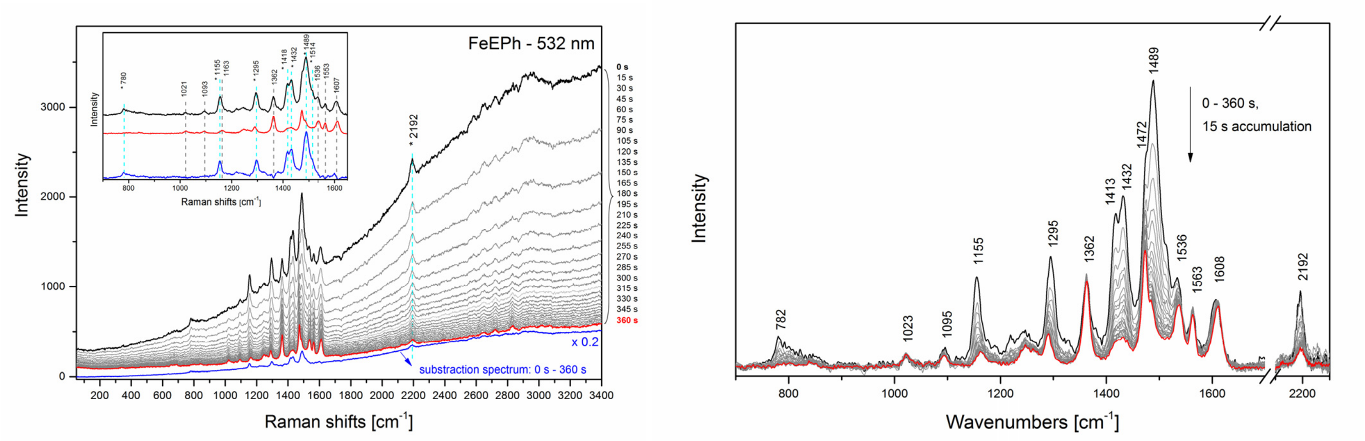

Figure S9. Left: Raman spectral set for **FeEPh** (laser power **0.1 mW**); INSET: **black**: starting spectrum; **red**: spectrum after 360 s; **blue**: difference spectrum **black** – **red**. Right: The spectral set after subtraction of fluorescence background to better see changes in spectral pattern.

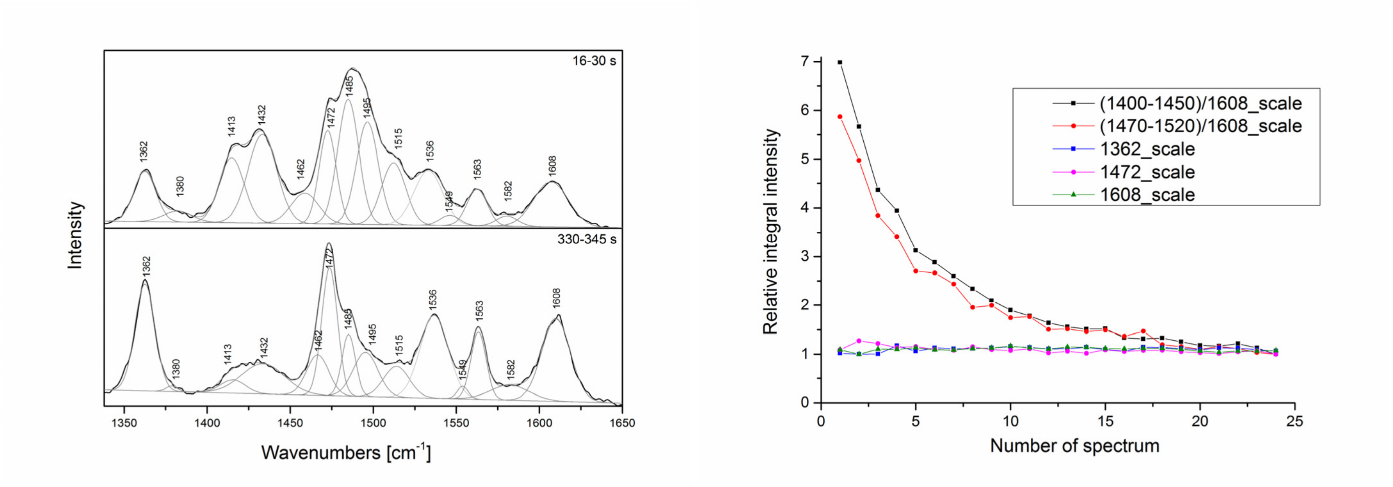

Figure S10. Deconvolution of the second and next-to-the-last spectrum of **FeEPh** and kinetics of obtained bands.

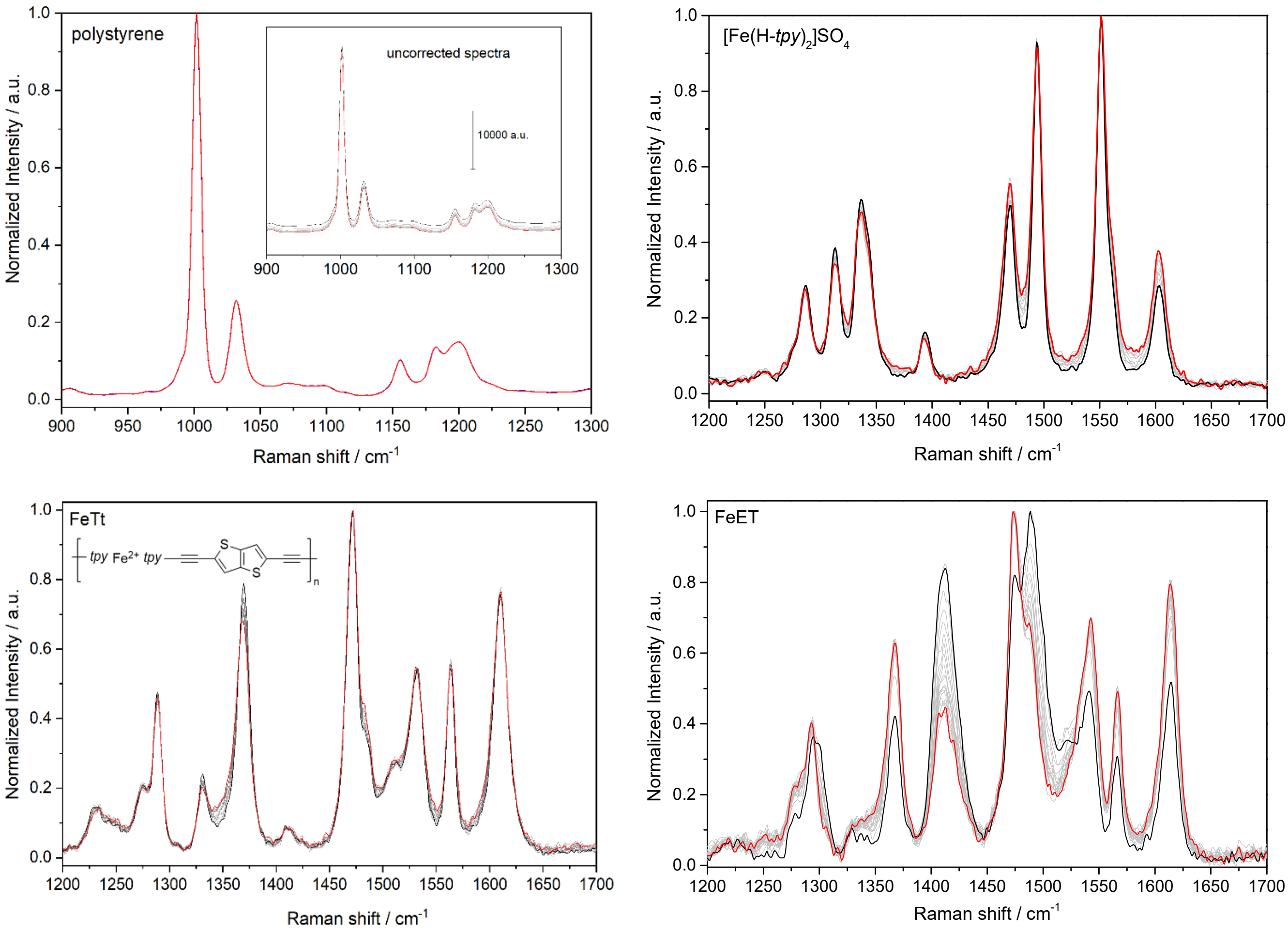

Figure S11 – Overlaid baseline corrected spectra of the spectral sets of indicated compounds, normalized to the highest spectral band; black – first measured spectrum (0 – 15 s), red – last measured spectrum (300 – 315 s).

The above figures demonstrate the reliability of the baseline corrections of spectra by the method of orthogonal differences. The uncorrected RS of polystyrene, thermally as well as photochemically stable material, provide identical RS after this correction. The resonance spectra of simple complex salt [Fe(H-*tpy*)$_2$]SO_4 and the 1-thioxophosphole-free polymer **FeTt** have shown a slight thermal broadening of Raman bands, whereas the RRS of **FeET** corrected in the same manner clearly showed convincing changes over time, which cannot be caused by baseline corrections.
